# The prevalence of deranged C-reactive protein and albumin in patients with incurable cancer approaching death

**DOI:** 10.1371/journal.pone.0193693

**Published:** 2018-03-13

**Authors:** Sarah Gray, Bertil Axelsson

**Affiliations:** 1 Research and Development Unit, Östersund Hospital, Östersund, Sweden; 2 Department of Radiation sciences, Unit of Clinical research—Östersund, Umeå University, Umeå, Sweden; University of South Alabama Mitchell Cancer Institute, UNITED STATES

## Abstract

**Introduction:**

Amongst patients with incurable cancer approaching death, cachexia is common and associated with adverse outcomes. The term cachexia lacks a universally accepted definition and there is no consensus regarding which variables are to be measured. Furthermore, an elevated C-reactive protein is a common clinical challenge in this patient group. This study aims to add to the ongoing discussion regarding the definition of cancer cachexia and to study the role of C-reactive protein and s-albumin in this context.

**Material and methods:**

A 1-year cohort, consisting of 155 cancer patients enrolled in a specialized palliative home care team in the city of Östersund, Sweden, that were deceased during the year of 2015 was studied. Laboratory measures were studied within 0–30 and 31–60 days prior to death. C-reactive protein >10 mg/L and coinciding s-albumin <30 g/L was referred to as “laboratory cachexia”. Also, the number of days from the first found “laboratory cachexia” until death was noted.

**Results:**

The prevalence of “laboratory cachexia” was 85% 0–30 days prior to death compared to 66% 31–60 days prior to death *(p<0*.*01)*. The majority of patients (75%) had an onset of “laboratory cachexia” within 0–120 days prior to death, with a median of 47 days. The median values for C-reactive protein and s-albumin within 0–30 days prior to death were 84mg/L and 23g/L respectively.

**Discussion:**

Could markedly deranged values of C-reactive protein and s-albumin, such as found in this study, signal a relatively short remaining survival time in patients with incurable cancer and no clinical signs of ongoing infection? The role of “laboratory cachexia” in this context as well as the cut off values for the laboratory measures included may be further discussed.

## Introduction

Cancer is a major cause of death and suffering worldwide. In Sweden it is responsible for approximately 21 000 deaths each year, which accounts for nearly 25% of all deaths [1, 2].

Amongst patients with advanced cancer the multifactorial syndrome of cancer cachexia [3] is common and has been estimated to affect up to 50–80% depending on the definition being used [4]. It is characterised by an ongoing loss of skeletal muscle mass (with or without loss of fat). The underlying pathophysiology consists of negative protein and energy balance driven by a variable combination of reduced food intake and abnormal metabolism, due to direct effects of tumour metabolism, systemic inflammation and other tumour mediated effects [3, 5, 6]. It is associated with decreased physical function and shortened survival [5, 7], however the term lacks a universally accepted definition and there is no true consensus regarding which variables are to be measured [8]. The condition is generally defined as a certain percentage of weight loss (2–20%) over a certain period of time (3–12 months), elevated C-reactive protein (CRP) >5–10 mg/L and lowered s-albumin (s-alb) <32–35 g/L are sometimes included as”minor criteria”[8–12]. The latest stage of cachexia is referred to as refractory cachexia, a stage with a duration of survival less than 3 months that cannot be reversed by conventional nutritional support and that is unresponsive to anticancer treatment [3]. This stage however is not easily distinguished from preceding stages clinically. Since we cannot yet discriminate between the different stages of cachexia, the wider term “cancer cachexia” is used in this study to cover all the stages of cachexia when referring to the syndrome.

In the advanced stages of disease accurate prognostication is particularly important in aiding clinical decision making as well as providing better possibilities for patients and families to plan and prepare for the time ahead [13]. Clinically it would be especially valuable to find a way to identify incurable patients with short survival prognosis, as this would aid in avoiding over-treatment with extensive surgery or palliative chemotherapy in patients likely to die before experiencing any advantageous effects. Ways of prognosticating survival in advanced cancer patients have been assessed and many proposals made [14–18]. As cancer cachexia is associated with shortened survival, many of its features are involved in these tools for prognostication. So far the most accurate methods suggested involve performance status (PS) alone or in combination with different scoring models such as the Palliative prognostic score (PaP-score) and the Palliative prognostic index (PPI) [13, 16, 19], whereas Clinician predicted survival (CPS) has shown to be rather inaccurate and tends to overestimate survival time [20]. The modified Glasgow prognostic score (mGPS) which uses CRP and albumin concentrations alone, has demonstrated a prognostic power in line with that of PS [18] and if combined with PS its accuracy in predicting a 3-month survival is increased even further. This suggests that an elevated CRP and decreased s-alb could be of use in terms of avoiding over-treatment as mentioned above, however deranged laboratory values in this patient group still remain a clinical challenge as they often cannot distinguish infected from non-infected patients [21].

At this time, there is not sufficient evidence that prognostication using above mentioned models is accurate enough for having a true impact in clinical practice when handling individual patients [14, 17, 22, 23]. Further investigation is needed to determine if and how these laboratory measures and scoring systems should be used in the future for more accurate prognostication. Further exploration of the cancer cachexia syndrome, its prevalence and the temporal association between its onset and death is also desirable. For this to be possible a more precise definition of the syndrome is required.

This study aims to add to the ongoing discussion regarding the definition of cancer cachexia syndrome and more specifically to study the role of CRP and s-alb in this context. CRP>10mg/L and coinciding s-alb <30g/L will be referred to as “laboratory cachexia”. Primary question: What is the prevalence of “laboratory cachexia” in a population of patients with incurable cancer approaching death? Secondary questions: What are the median values of CRP and s-alb during the last two months of life? When in relation to death is the criteria of "laboratory cachexia" fulfilled for the first time? How many cases are excluded if the criteria for "laboratory cachexia" are adjusted? Our hypothesis is that these laboratory measures that could imply the presence of cancer cachexia will be altered in a majority of the study population during the last months of life.

## Material and methods

A 1-year cohort, consisting of patients who were enrolled in a specialized palliative home care team (SPHCT) in the city of Östersund, Sweden and deceased during the year of 2015 was studied retrospectively. All adult patients with a cancer diagnosis were included. For each patient laboratory measures including haemoglobin (Hb), CRP, leukocytes (WBC), creatinine (crea), s-alb, corrected calcium (corr Ca) and sodium (Na) were assessed within two separate time periods. These were 0–30 days (0-30d period) and 31–60 days (31-60d period) prior death, if more than one measurement was available within given period, the value closest to the date of death was used. CRP >10 mg/L and coinciding s-alb <30 g/L was used to determine the presence of “laboratory cachexia”. In addition to this, the number of days from the first found “laboratory cachexia” until death was noted for each patient (first found “laboratory cachexia” meaning that this was the first time the criteria were fulfilled and were continuously fulfilled in all available measurements from then on). Medication with steroids (betamethasone, prednisolone) was also recorded separately for both time periods, as they could have an impact on the laboratory measurements. None of the patients received chemotherapy during the time period studied.

### Ethics

The study was performed in accordance with the ethical standards laid down in the 1964 Declaration of Helsinki and its later amendments and was approved by the ethical committee of Umeå university, registration number 2016-496-32M. Informed consent was not obtained from families or caregivers of the deceased patients in this retrospective study as this requirement was waived by the ethical committee.

### Statistics

The received data was primarily processed descriptively. Differences between groups were determined using below mentioned tests. Wilcoxon signed ranked test and Mann Whitney U was used for continuous variables when comparing two groups and Chi Squared test for categorical variables. A p-value <0,05 was considered statistically significant. When values within the different time periods were compared, only patients with available values for that specific measure within both time periods were included in the analyse. The exact numbers are specified below. All statistics were performed with the StatView statistical program for windows, version 5.0.1 (SAS institute, cary, NC, USA).

## Results

### Study population and descriptive information

**Fig 1:**
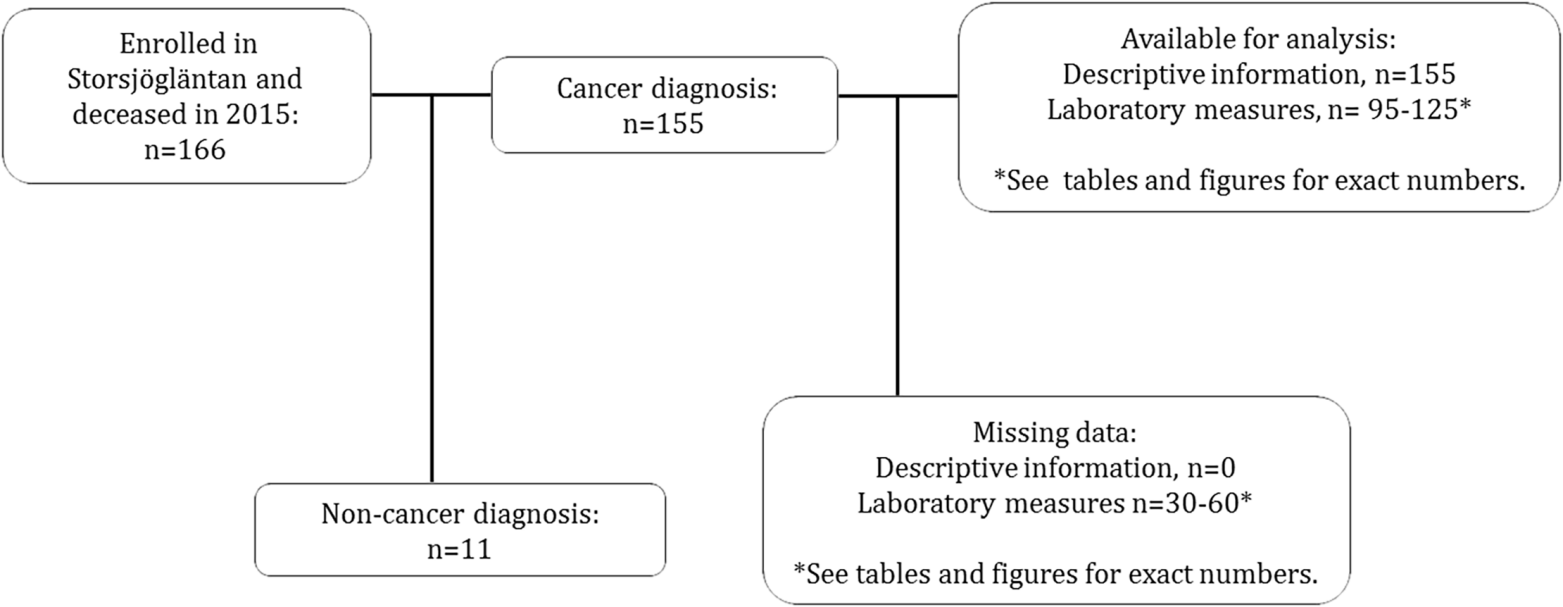
Flow of selection process.

One hundred sixty six patients enrolled in the SPHCT were diseased in the year of 2015, out of them 155 had a cancer diagnosis. The selection process can be viewed in Fig 1. The study population as a whole had a median age of 72 years (range 23–94) and 57% were males. The most common tumour types were upper gastrointestinal (n = 41), urological or gynaecological (n = 35) and lower gastrointestinal (n = 18), the remainder of diagnoses was made out by lung, breast and other or unspecified tumours. Descriptive information is presented in Table 1.

**Table 1:** Patient characteristics at baseline.

	Median	N(%)
Patients		155
Age, years (Range)	72 (23–94)	
**Sex**		
Male		88(57)
Female		67(43)
**Tumour type**		
Lung		16(10)
Breast		11(7)
Upper GI		41(26)
Lower GI		18(12)
Urological/Gynecological		35(23)
Other/unspecified		34(22)
**Ongoing steroid medication**		
Last month of life (0-30d)		134(86)
2nd last month of life (31-60d)		98(63)

### Laboratory measures and “laboratory cachexia”

**Fig 2:**
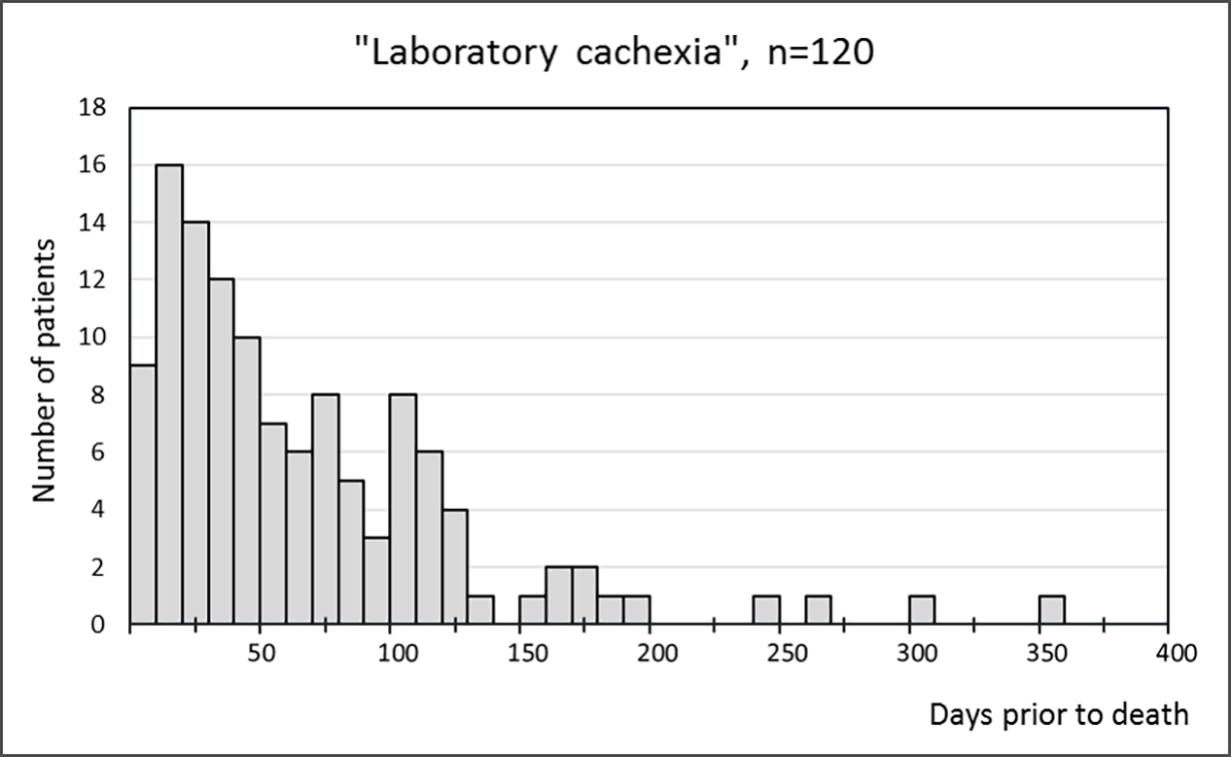
Laboratory cachexia. Number of days prior to death that the criteria for “laboratory cachexia” was first fulfilled for the 120 patients who at some point fulfilled the criteria.

For CRP, WBC, s-alb, Corr Ca and Na, median laboratory values were significantly different within the two time periods. Within the 0-30d period values for CRP and s-alb were as follows: CRP = 84mg/L (113 IQR) and s-alb = 23 g/L (8 IQR) whilst values within the 31-60d period were for CRP, 44 mg/L (87 IQR) and s-alb 26 g/L (8 IQR). Significant differences were not found for Hb and Crea, all median values including p-values are presented in Table 2. Looking at only those with values for CRP and s-alb within both time periods, N = 95, a total of 81 (85%) fulfilled the criteria of laboratory cachexia within the 0-30d period and 63 (66%) within the 31-60d period, this is presented in Table 3. When instead looking at those who at some point fulfilled the criteria of laboratory cachexia and then continuously fulfilled the criteria in all available measurements from then on, N = 120, the median number of days from first found CRP >10 mg/L and coinciding s-alb <30 g/L was 47days. This is shown in Fig 2. A majority of 104 (75%) had fulfilled the criteria within 0–120 days prior to death and 16 (12%) fulfilled the criteria more than 120 days prior to death. Out of those with available measurements for CRP and s-alb, 18 (13%) never fulfilled the criteria and are therefore not included in the figure. Those who never fulfilled the criteria of “laboratory cachexia” did not differ from the rest of the study population in regards of age, gender, tumour type or medication.

**Table 2:** Median laboratory values (ICR).

Biochemical measure	N	0–30 days	31–60 days	P-value
Haemoglobin, g/L	125	112 (27)	111 (19)	NS (0,74)
CRP, mg/L	96	84 (113)	44 (87)	<0,01
Leukocytes, 10e9/L	125	11,0 (8,1)	9,6 (6,8)	<0,0001
Creatinine, μmol/L	119	67 (50)	66 (28)	NS (0,56)
Albumin, g/L	120	23 (8)	26 (8)	<0,0001
Corrected Calcium, mmol/L	120	2,37 (0,25)	2,42 (0,24)	<0,01
Sodium, mmol/L	120	136 (8)	137 (5)	<0,05

Median laboratory values (IQR) during the last month of life (0–30 days) and the 2nd last month of life (31–60 days). For each biochemical measure, only patients with values within both time periods are included.

**Table 3:** “Laboratory cachexia”.

	0–30 days, n = 95	31–60 days, n = 95	
	N(%)	N (%)	P-value
yes	81(85)	63(66)	<0,05
no	14(15)	32(34)	

“Laboratory cachexia” (CRP >10 and albumin <30) during the last month of life (0-30days) and the 2^nd^ last month of life (31–61 days). Only patients with available measurements for both values within both time periods were included.

**Fig 3:**
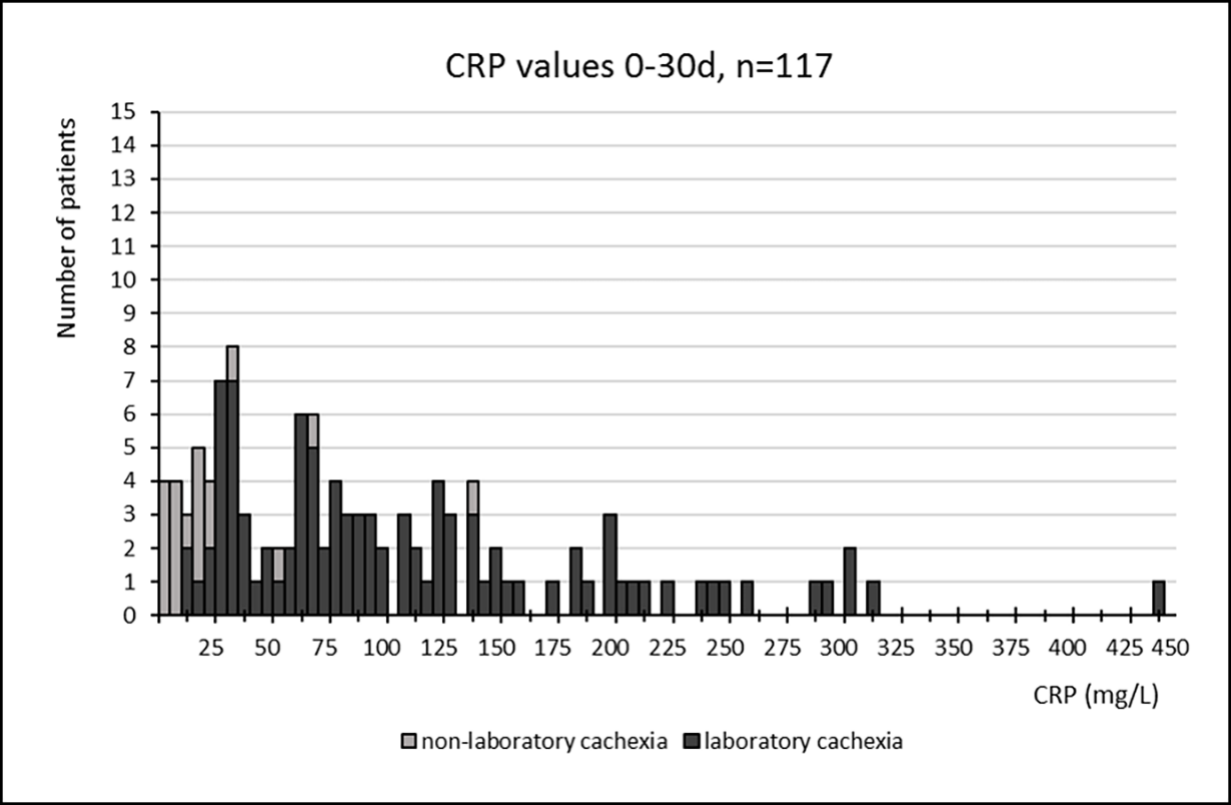
CRP values, 0-30d. CRP values within the 0-30d period for all patients with available measurements for both CRP and s-albumin within this period.

**Fig 4:**
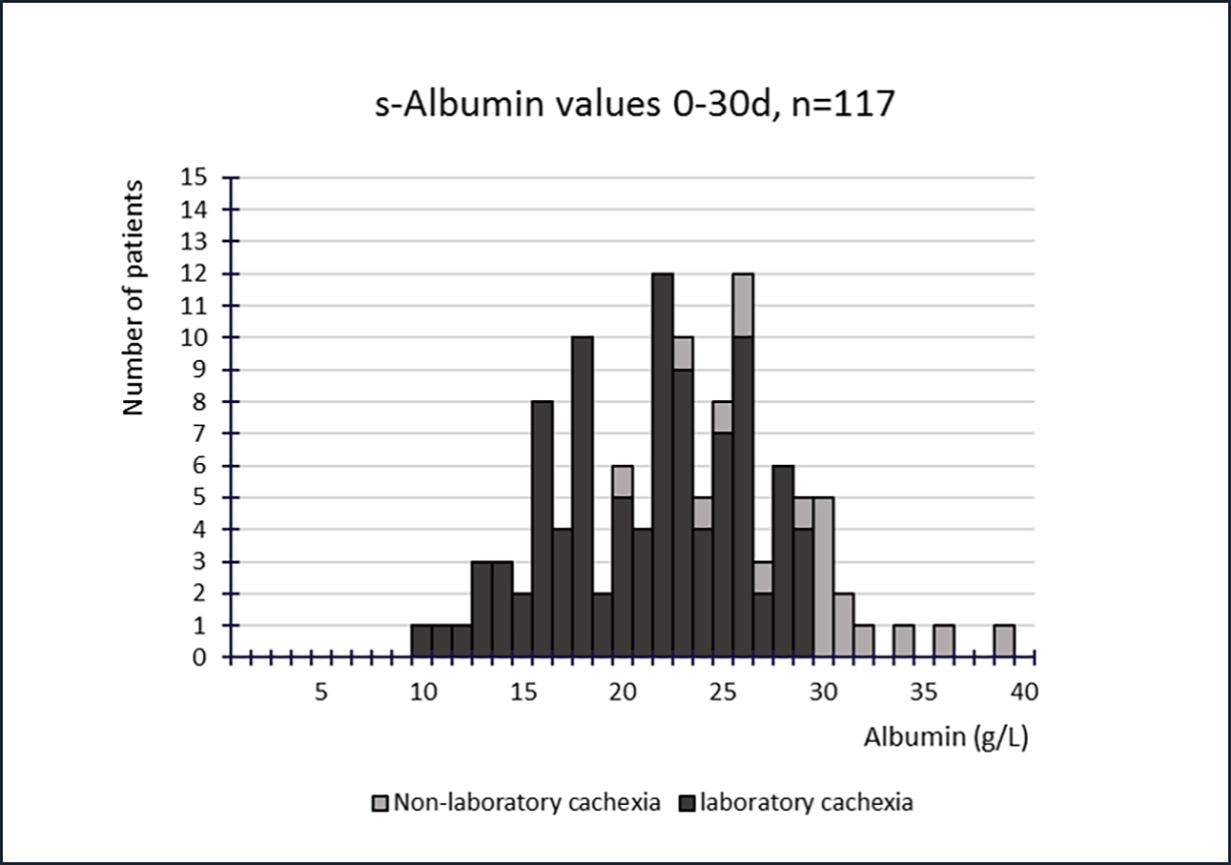
S-albumin values, 0-30d. S-albumin values within the 0-30d period for all patients with available measurements for both CRP and s-albumin within this period.

Figs 3 and 4 show CRP and s-alb values respectively for all patients within the 0-30d period with available data for both values. Both figures allow us to distinguish between those who fulfilled the criteria of laboratory cachexia from those who did not. If the cut off for CRP was reformed to >25 instead of >10, an additional 5(4%) would be excluded from fulfilling the criteria, however if the cut off for s-alb was set to <25 instead of 30, an additional 29(25%) would be excluded. If the cut off for albumin instead was changed to <32, an additional 7(6%) would be instead included.

## Discussion

In accordance with our hypothesis, laboratory measures that imply the presence of cancer cachexia were altered in the majority of patients during the last month of life. The criteria of “laboratory cachexia” was fulfilled in 85% of patients within the 0-30d period compared with 66% in the 31-60d period. The median for first found CRP >10mg/L and s-alb <30g/L was 47 days prior to death, a markedly increase in number of patients showing these alterations in CRP and s-alb was seen from roughly 120 days prior to death and onwards.

We also discovered remarkably deranged median values for CRP and s-alb in the group as a whole. The median CRP value was particularly high in the last month of life (84mg/L) and a more thorough investigation showed that only 5 cases (4%) were excluded from our criteria of “laboratory cachexia” within the 0-30d period if the criteria for CRP was altered to include only values >25mg/L. The median value for s-alb was rather low (23g/L), yet 29 cases (25%) would be lost if the cut off was altered for s-alb to include only values <25g/L and an additional 7(6%) would be included if the cut off was instead changed to <32. These results suggest that the cut off at <30g/L for s-alb is already somewhat strict, however for CRP the question arises whether or not there is a potential gain in altering the cut off to >25mg/L instead of >10mg/L.

Depending on the type of cancer and the definition being used for cachexia prevalence estimates range from 50–80% in advanced cancer patients [4]. Although we cannot compare our results directly due to different definitions being used, our results based on “laboratory cachexia” propose a prevalence of 84%, not far from above mentioned range. This could to some extent enforce that our results are reasonable. To our knowledge, no studies investigating the time of onset of the cancer cachexia syndrome in relation to death have been made. This study presents a median of 47 days for “laboratory cachexia” and that the onset based on these criteria lies within 0–120 days prior to death for the majority (75%) of patients.

As was discussed in the introduction, it is difficult to separate the different stages of cancer cachexia clinically. However, seeing as we have studied patients in their last months of life, it is likely that it is the refractory stage being brought to light, although we cannot state this for sure. Nevertheless, our results show an onset of “laboratory cachexia” 0–120 days prior to death in the majority of patients. This may be considered to be somewhat in line with the maximum survival time of 3 months for refractory cachexia as was proposed by Fearon et al. [3].

The term “laboratory cachexia” has not previously been used and no information regarding presence of weight loss, anorexia or fatigue was available in this study. Therefore no correlation between “laboratory cachexia” and presence of cachexia according to current consensus definition could be done [3]. However, CRP and s-alb can be considered valid markers of systemic inflammation and also as a part of the criteria for cancer cachexia [24]. As mentioned above there is no consensus regarding the cut offs for these measures, however >5–10 for CRP and <32–35 for s-alb have been used in other studies [12, 18, 25, 26]. The cut offs used in this study may therefore be considered to be relatively strict, furthermore the results also propose that the cut off for CRP could potentially be made even more strict, as CRP values in general were found to be remarkably elevated.

Although many studies involve palliative cancer patients, to our knowledge no other study has specifically studied CRP and s-alb values in patients during their last 2 months of life. This could perhaps explain why the laboratory measures in this study population were more deranged than what has been seen in other studies [5, 12, 27]. A correlation between CRP and survival has been found in previous studies [28, 29]. Based on the wide spread in CRP and s-alb values, perhaps scoring models such as the mGPS could be further modified, which could potentially improve the accuracy of survival prediction. However, this would need to be confirmed in a new set of studies before any such conclusions can be drawn.

The aim of this study was to focus on CRP and s-alb values in the clinical context of end-of-life care. The reason behind choosing to study these measures over other systemic markers such as cytokines interleukin 6 (IL6) and interleukin (IL1), which are not routinely analysed, was primarily the accessibility of CRP and s-alb values from medical records in a retrospective setting.

### Limitations

A limitation of this study is its retrospective design as it involves the disadvantage of lacking data to some extent. In this study “laboratory cachexia” was used as a surrogate marker for cancer cachexia, as data for weight loss was not available. However, the accuracy of the estimated prevalence of cancer cachexia based on the prevalence of “laboratory cachexia” is uncertain.

Our usage of CRP in the criteria for “laboratory cachexia” can be considered problematic as CRP is not specific for systemic inflammation. However, all patients in this study were under specialised palliative care where appropriate treatment of infections may be assumed to have been given when relevant. Therefore, we consider the majority of elevated CRP values to be a result of the underlying cancer even though it cannot be completely ruled out that infection may be the accountable reason in some of the cases.

The wide spread in the onset of “laboratory cachexia” and the fact that not all patients fulfilled the criteria must also be taken into consideration when evaluating its practical usefulness.

### Implications

The implication of our findings is a stricter cut off for CRP when defining cancer cachexia than what has previously been suggested. The cut off for s-alb may also be discussed, as the cut off used in this study (<30g/L) was slightly lower than what other studies have proposed. This opens up for an extended discussion regarding the definition of cancer cachexia, the proposed cut offs for the laboratory measures included in this definition and their future use. For instance, should deranged CRP and s-alb values in the absence of clinical signs of infection, be interpreted as a sign of short survival and thus play a larger role in clinical decision-making regarding whether or not to continue treatment such as palliative chemotherapy and extensive surgery? To further help distinguish between systemic bacterial infection and inflammation related to cachexia, inclusion of procalcitonin (PCT) in addition to CRP and s-alb may be of value in clinical practice and also future studies.

### Conclusions

Laboratory measures that imply the presence of cancer cachexia (CRP and s-alb) had a median onset of 47 days prior to death and during the last month in life were altered in 85% of the patients. Values for CRP and s-alb was altered to such an extent during the last month of life that they, in the absence of clinical signs of infection, may be used as a complementary indication of short remaining life time. Further investigation is needed to determine the role of “laboratory cachexia” in the context of cancer cachexia, as well as the exact definition and classification of the cancer cachexia syndrome itself.

## Supporting information

S1 Dataset (XLSX)
